# On the Changes Which Ether, Alcohol, and Bodies of a Similar Constitution, Suffer, When Taken into the Circulation

**Published:** 1850-08

**Authors:** Charles W. Wright

**Affiliations:** Cincinnati


### CHEMISTRY.

*On the changes which Ether, Alcohol, and bodies of a similar constitution, suffer when taken into the Cirulation.* By Charles W. Wright, M. D., of Cincinnati.—The rapidity with which certain substances manifest their action when taken into the circulation, has often engaged the attention of medical men, and more especially so since the discovery of the remarkable anæsthetic properties of the ethers, naphtha, and chloroform.

It is observed that when a medicinal substance is inhaled, much less is required to produce its characteristic effects than when it is administered by the mouth; and it is often stated that therapeutic agents operate quite differently when taken into the stomach, from what they do when inspired. This difference seems to be more apparent than real, as I think a thorough examination into this subject would plainly show; and if there be a difference, it is more in degree and rapidity than in kind.

Ether and alcohol are the first that will be considered under this head.

The first effect of the inhalation of the vapor of ether is to stimulate the system powerfully, but this state of excitement soon passes off, and is rapidly succeeded by a lethargic condition of the system, the skin becoming cold and pale, lips of a livid hue, slow and laborious respiration, in fact coma supervenes, and this in from two to five minutes. Introduce the same substance, but in larger quantity, in the liquid form, into the stomach, and what then occurs? why the same train of symptoms precisely, but which are much slower in making their appearance, and which are protracted a greater length of time. That it should require a larger quantity of ether to produce these effects through the medium of the stomach than the air passages, is obvious, for when ether, in the form of vapor, is enhaled, being exposed to a great extent of absorbing surface, it passes at once into the circulation, and there meeting with oxygen in a very active form, enters into combination with it, forming carbonic acid and water, being attended at first with excitement which retards the supervention of coma. The combustion of the ether is very rapid at first, but as the oxygen is consumed it fails to perform its task of purifying the blood, the consequence of which is, carbonic acid accumulates in that fluid, and death is finally brought about in precisely the same manner as if the patient had in the first instance inhaled carbonic acid gas. The impression which I wish to convey may be illustrated by comparing the inhalation of ether to the common experiment of plunging a lighted taper into a vessel of oxygen gas. In that case the combustion begins with great energy, but the flame grows less and less, until it is finally extinguished. Now there are two causes in operation tending to retard and ultimately arrest the combustion in this experiment; the first is the gradual disappearance of the oxygen from the vessel in which it is contained, the second is the influence which carbonic acid exerts in diminishing the combustion going on in flame.

The excitation of ether, when first administered, is very rapid; but as carbonic acid is generated in proportion as it is consumed, and the blood in other respects fails to be decarbonized, the excitement is soon succeeded by lethargy, the person dying as if poisoned by carbonic acid; the symptoms and post-mortem appearances being the same in both cases. In the former, the poison is formed by combustion going on in the system, but in the latter it is formed out of the system and inhaled.

The arrest of the circulation is not unlike that which results from the respiration of nitrous oxide gas, but from a different cause. When the exhilarating gas is inhaled a considerable length of time, the pulmonary capillaries no longer have an attraction for venous blood, it being saturated with oxygen. Carbonic acid produces just the opposite state, by charging the blood throughout the body with carbonic acid, and destroying the attraction which the systemic capillaries have for the blood in the arteries. Great irritability is induced by the former substance, and the heart continues to pulsate even after the circulation in other respects has ceased.

When taken into the stomach ether produces the same set of symptoms, with this difference, that the stage of excitement lasts longer, and if the quantity taken be not too large, gives the system time to throw it off in the form of water and carbonic acid. Even here, however, if an overdose be swallowed it may prove fatal, the symptoms being the same as when it is respired.

The symptoms ocasioned by the introdution of alcohol into the circulation, are much the same as those produced by ether. This will appear evident when we look at the composition of these two bodies. Alcohol is represented by the formula $C_4\ H_5 + O \cdot O$. Ether has the same constitution, $C_4\ H_5 + O$, minus one atom of water, H O.

When consumed, they both yield the same products. That of ether $12\ O + C_4\ H_5\ O4 = C\ O_2 + 5\ H\ O$. That of alcohol $12\ O + C_4\ H_5\ O + H\ O + = 4\ C\ O_2 + 6\ H\ O$.

Naphtha has been used as an anæsthetic agent, and it probably operates on the same principle, but from its producing a very rapid and fluttering pulse, its employment is not considered safe.

Carbonic oxide produces a pleasing delirium before it narcotizes, if gradually introduced into the lungs; but cases of poisoning with this gas are exceedingly rare.

That there are other influences operating here, I do not pretend to deny, but that the characteristic effects of these agents are produced in the manner set forth, I think a thorough examination of this subject in all its bearings would not fail to show.—*Western Lancet.*

